# Oxidative stress and antioxidant defense in detoxification systems of snake venom-induced toxicity

**DOI:** 10.1590/1678-9199-JVATITD-2020-0053

**Published:** 2020-10-19

**Authors:** Degang Dong, Zhongping Deng, Zhangren Yan, Wenli Mao, Jun Yi, Mei Song, Qiang Li, Jun Chen, Qi Chen, Liang Liu, Xi Wang, Xiuqin Huang, Wanchun Wang

**Affiliations:** 1 School of Life Sciences, Jiangxi University of Traditional Chinese Medicine, Nanchang, China.; 2 Innovative Chinese Medicine Research Institute, Shanghai University of Chinese Medicine, Shanghai, China.; 3 Southern Snake Bite Control Center, Affiliated Hospital of Jiangxi University of Traditional Chinese Medicine, Nanchang, China.; 4 Science and Technology College, Jiangxi University of Traditional Chinese Medicine, Nanchang, China.; 5 College of Chinese Medicine, Hunan University of Chinese Medicine, Changsha, China.

**Keywords:** Snake, Venom, Proteome, Hydrogen peroxide, Antioxidant defense

## Abstract

**Background:**

Snakebites remain a major life-threatening event worldwide. It is still difficult to make a positive identification of snake species by clinicians in both Western medicine and Chinese medicine. The main reason for this is a shortage of diagnostic biomarkers and lack of knowledge about pathways of venom-induced toxicity. In traditional Chinese medicine, snakebites are considered to be treated with wind, fire, and wind-fire toxin, but additional studies are required.

**Methods:**

Cases of snakebite seen at the Affiliated Hospital of Jiangxi University of Traditional Chinese Medicine were grouped as follows: fire toxin - including four cases of bites by *Agkistrodon acutus* and three bites by *Trimeresurus stejnegeri* - and wind-fire toxin - four cases of bites by vipers and three bites by cobras. Serum protein quantification was performed using LC-MS/MS. Differential abundance proteins (DAPs) were identified from comparison of snakebites of each snake species and healthy controls. The protein interaction network was constructed using STITCH database.

**Results:**

Principal component analysis and hierarchical clustering of 474 unique proteins exhibited protein expression profiles of wind-fire toxins that are distinct from that of fire toxins. Ninety-three DAPs were identified in each snakebite subgroup as compared with healthy control, of which 38 proteins were found to have significantly different expression levels and 55 proteins displayed no expression in one subgroup, by subgroup comparison. GO analysis revealed that the DAPs participated in bicarbonate/oxygen transport and hydrogen peroxide catabolic process, and affected carbon-oxygen lyase activity and heme binding. Thirty DAPs directly or indirectly acted on hydrogen peroxide in the interaction network of proteins and drug compounds. The network was clustered into four groups: lipid metabolism and transport; IGF-mediated growth; oxygen transport; and innate immunity.

**Conclusions:**

Our results show that the pathways of snake venom-induced toxicity may form a protein network of antioxidant defense by regulating oxidative stress through interaction with hydrogen peroxide.

## Background

Venomous snakebites have been a common and critical clinical illness since ancient times [1]. The popular story about the farmer and the snake from Aesop’s Fables illustrates that fatal snake bites were one of the major public health challenges. The World Health Organization estimated that about 4.5 to 5.4 million snake bites occur globally each year, resulting in 1.8 to 2.7 million cases of snakebite envenomings and 81,000 to 138,000 deaths [2]. Snakebites frequently occur in tropical, developing countries in South Asia, South-East Asia, Africa and Latin America [3-6]. Within China, there are approximately 200 species of snakes, including about 50 species of poisonous snakes and more than 10 species of highly virulent snakes [7]. Bites by dangerous snakes are greatly feared, and effective symptomatic diagnosis and treatment are poorly documented. Due to complex pathogenesis and interspecific variability of venom components, the mechanisms and pathophysiological changes are still not completely elucidated [8, 9]. 

Symptoms induced by snake venoms are multi-factorial and complex, which severely restricts therapeutic intervention after the snake bite [10]. Moreover, differing components of different venoms make it difficult to generate sufficient diagnostic markers for clinical practice in both Western medicine and Chinese medicine [11, 12]. Quantitative proteomic analysis of multiple venoms revealed that the venom proteome composition includes non-enzymatic and enzymatic proteins, such as three finger toxins (3FTx) and phospholipase A_2_ PLA_2_ [13-15]. In the absence of sufficient diagnostic markers, the clinical identification of a venomous snake is mainly based on the patient's wound tooth distance, tooth marks, local symptoms, and consultations. Traditional Chinese medicine (TCM) classifies venomous snakes on the basis of toxicity symptoms into three types: wind toxin, fire toxin, and wind-fire toxin [16]. The fire toxin is similar to hemorrhagic venom. Clinical manifestations of snakebites by fire toxin snakes include local pain, immediately swelling, water or blood blisters around the bite on the affected limb, skin ecchymosis, ulceration, necrosis, and general hemorrhage. The wind toxin can be regarded as part of neurotoxic venoms, generating mild pain, minor blood loss, numbness, somnolence, difficulty in opening the mouth, confusion, respiratory distress, blurred vision, etc. The wind-fire toxin is similar to mixed venom, which presents a combination of neurotoxic and hemorrhagic symptoms. The wind-fire toxin provokes respiratory distress, drooping eyelids, blurred vision, muscle weakness, and paralysis of muscles in the extremities. In our TCM clinical work, fire toxin and wind-fire toxin are particularly prevalent, so that the predominant venomous snake species in China is deserving prior attention, including *Agkistrodon acutus* and *Trimeresurus stejnegeri* snakebite (fire toxin), and viper and cobra snakebite (wind-fire toxin). In TCM, the same treatment strategy is implemented for the same snakebite syndrome, even in different patients that were bitten by different snakes. At present, the identification of the snakebite syndrome in TCM relies on expert judgment and knowledge, such as diagnosis by exclusion, and speculative and empirical judgments. The emergency treatment of snakebites is based primarily on clinical practice guidelines, some of which lack scientific study. Furthermore, our understanding of the relationship between Compound Chinese Medicine and the effects of multiple components is still in its infancy [17]. Rapid and accurate identification of the snakebite etiology is also an important prerequisite for timely and effective treatment in TCM. It is urgent to correlate the pharmacological properties to clinical manifestations for a better understanding of wind-fire toxin and fire toxin. Investigation and analysis of the protein composition of serum from snakebite patients for diagnostic biomarkers will contribute to the clinical practice. 

This study aims at solving diagnostic markers and investigating pathways of snake venom-induced toxicity from serum proteins of snakebite patients with four predominant venomous snake species in China. The serum protein profiles reflected the functional categorization. The differential abundance proteins (DAPs) supplied discovery biomarkers to distinguish snakebite by species. The network of interacting proteins and drug compounds contribute to understanding the complex pathogenesis of venom-induced toxicity. Serum proteome analysis could contribute to TCM diagnostic markers of snakebite syndromes, providing clinicians with a clear distinction between the wind-fire toxin and fire toxin, as well as early clinical identification of snake species.

## Materials and Methods

### Patient identification and eligibility

The experimental procedures were approved by the Ethics Committee of the Affiliated Hospital of Jiangxi University of Traditional Chinese Medicine (n. JZFYLL2017103012). Informed consent forms were obtained from all recruited participants in the surgical department of the Traditional Chinese Medicine of the Affiliated Hospital. All identified snakebite patients were reviewed for eligibility to inclusion and exclusion criteria. Patients were excluded for the following reasons:

- received invasive examinations and/or treatments within three months of snakebite;
- treated with antivenom serum after snakebite;
- subjected to antitumor immunotherapy;
- diagnosed with liver or kidney disease affecting serum proteomics.

Clinicians identified the pattern of fire toxin and wind-fire toxin according to the standards of TCM, including clinical diagnostic experience and patient medical records. 

### Sample preparation and patient selection

From the serum samples available, we selected four cases bitten by vipers (B) and three cases bitten by cobras (C) as the wind-fire toxin group; four cases bitten by *Agkistrodon acutus* (D) and three cases bitten by *Trimeresurus stejnegeri* (E) as the fire toxin group; and fifteen healthy volunteers as the control group. The serum samples from 5 healthy controls were pooled together, so that the individuals in the control group were divided into three experimental duplicates, named A1, A2, and A3. The wind-fire toxin group was coded B1, B2, B3, B4, C1, C2, and C3; while the fire toxin group was coded D1, D2, D3, D4, E1, E2, and E3.

### Protein separation and LC-MS/MS analysis

All of the serum samples were processed as described previously [18]. Briefly, high abundance proteins were first depleted by multiple affinity removal according to the Agilent protein purification protocol [19]. Then, each depleted protein sample was separated on 12% gradient tris-glycine polyacrylamide gel using the sodium dodecyl sulfate polyacrylamide gel electrophoresis (SDS-PAGE) method [20]. The gel was stained with Coomassie blue, then re-suspended with dithiothreitol and digested with the sequence grade modified Trypsin (Promega) [21]. The digested proteins were passed through a C18 cartridge to be desalted. Finally, the dried peptides were resuspended in 40µL of 0.1% formic acid prior to LC-MS/MS. Then 2 µL peptide injection volume was loaded via a direct inject column configuration onto an Easy-nano LC 1200 instrument (Thermo Fisher Scientific) coupled online to a Q-Exactive Plus (Thermo Fisher Scientific). The resuspended peptides were first separated on Thermo Acclaim PepMap RSLC nanoViper C18 column (50 µm×15 cm) using solvent A (0.1% formic acid in water) and solvent B (80% acetonitrile in water containing 0.1% formic acid) as mobile phases for gradient elution at an auto-injector temperature of 30°C and a flow rate of 300 nL/min for 120 min [22]. Peptide elution was initiated with sustaining 6% solvent B (80% acetonitrile with 0.1% formic acid) for minutes 0-5, ramping solvent B 6-28% for minutes 5-105, ramping solvent B 28-38% for minutes 105-110, ramping solvent B 38-100% for minutes 110-115, and maintaining solvent B at 100% for minutes 115-120. The eluted peptides were introduced to the mass spectrometer and a full MS scan was acquired in the m/z range of 350-1,800 with a mass resolution of 70,000 at m/z 200 in the positive ion mode. The top 10 most intense peaks with a charge state ≥2 were fragmented with normalized collision energy 27 and isolation window 2 m/z. Tandem mass spectra were generated with a mass resolution of 17,500 at m/z 200. The maximum ion accumulation times were 50 ms and 45 ms for the survey scan and the MS/MS scans, respectively.

### Protein identification and quantitative analysis

The LC-MS/MS data were analyzed using Andromeda and MaxQuant (v 1.3.0.5) for peptide identification and quantification [23, 24]. The UniProt human isoform protein database was downloaded in August 2017, and contained 159,615 entries at this time. For the searches, the first search and mass tolerance of 20 ppm and two max missed cleavages were allowed. Fixed modification was set to carbamidomethyl (C), while variable modifications were oxidation (M) and acetylation (protein N-terminal). A false discovery rate (FDR) of 0.01 was set to filter the results at the peptide and protein levels. Matching and alignment time window in match between run options were set to 0.7 and 2 minutes, respectively. Furthermore, only unique proteins identified with more than unique razor peptides were considered [24]. Finally, a label-free quantification (LFQ) approach was carried out for normalization of protein intensities across runs with a minimum of one ratio count [23]. The LFQ measuring intensities were assigned to the relative abundance for each protein and used in the subsequent statistical analysis.

### Bioinformatics analysis

For the statistical analysis, *p*-values were calculated by Student’s t-test in R 3.5.0 under the null hypothesis to identify differentially expressed proteins between the two groups. At least two non-null values of data in each group were required for calculation of fold change and *p*-values. Proteins with expression changes with a *p*-value < 0.05 and |log2fold change| > 1 were selected as significant candidates. In addition, we took into account a comparison of all missing values in one group with expression values in another group. Proteins that were expressed in one group and not expressed in another group were considered to have a significant difference between the two groups.

For the functional annotation of identified proteins, the Gene Ontology (GO) and UniProt databases were used. GO enrichment analyses of differentially expressed proteins were performed with Fisher's exact test. An adjusted *p*-value <0.05 was chosen as the threshold for significance. A heat map of hierarchical clustering of expression proteins was generated using the web tool ClustVis (https://biit.cs.ut.ee/clustvis/) [25]. Protein-protein interaction (PPI) networks were conducted using the STITCH 5 database [26, 27]. 

## Results

### Identification of serum proteins by LC-MS/MS

Label-free quantitative proteomics of serum samples was performed on samples from 14 subjects with four types of snakebites and 15 healthy controls. Of the 14 patients, 7 were diagnosed by TCM to have wind-fire toxin including 4 snakebites by vipers (B) and 3 snakebites by cobras (C), and 7 were diagnosed by TCM to have fire toxin including 4 snakebites by *Agkistrodon acutus* (D) and 3 snakebites by *Trimeresurus stejnegeri* (E). The fifteen healthy controls were pooled into three samples as described in the methods. After preprocessing LC-MS/MS runs, the peptide intensities were obtained in MaxQuant analysis (Additional file 1). A total of 735 proteins with one or more unique peptides were quantified by LC-MS/MS analysis (Additional file 2). The representative proteins for each group were comprised of 471, 514, and 680 proteins in healthy control, wind-fire toxin, and fire toxin groups, respectively. When we filtered the results to satisfy the condition of getting at least two non-null intensities in replicates, we identified 474 unique proteins between patients with snakebites and healthy control.

**Figure 1.**
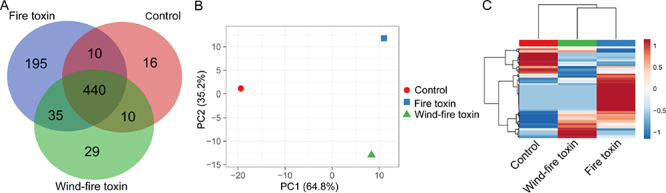
An overview of the distribution of the serum protein expression profiles. **(A)** A Venn diagram of the snakebite proteins in the wind-fire toxin and fire toxin groups and the healthy control group. **(B)** Principal component analysis accounted for 64.8% of the observed variation between the snakebite groups and the healthy control group at the first principal component (PC1), as well as 35.2% of variability between the wind-fire toxin and fire toxin groups at PC2. **(C)** The clustering of serum proteins revealed distinct protein expression patterns in the wind-fire toxin and fire toxin groups and the healthy control group.

We initially examined whether the serum proteins could discriminate between the two snakebite groups and the healthy control group. We identified 474 proteins in both patient groups, constituting 64.6% of the total proteins identified (Figure 1A). Based on the serum protein expression profiles, the three groups were differentiable by principal component analysis (PCA) (Figure 1B). We observed that the healthy control group was clearly separated from the bulk of snakebite patients at the first principal component (PC1), accounting for 64.8% of the observed variation. In contrast, the PC2 discriminated 35.2% of variability between the wind-fire toxin and fire toxin groups (Figure 1B). We next evaluated the hierarchical clustering, represented as a heat map of the relative protein abundance in each protein group. In the dendrogram, the clustering exhibited a similar expression trend across the wind-fire toxin and fire toxin groups, which appeared distinct from the protein expression patterns in the healthy control group (Figure 1C). This interpretation was consistent with the PCA results. Therefore, we conclude that the three main groups were clearly differentiable by their serum protein expression profiles.

### Differential abundance of proteins among the snakebite subgroups and the healthy control group

**Figure 2.**
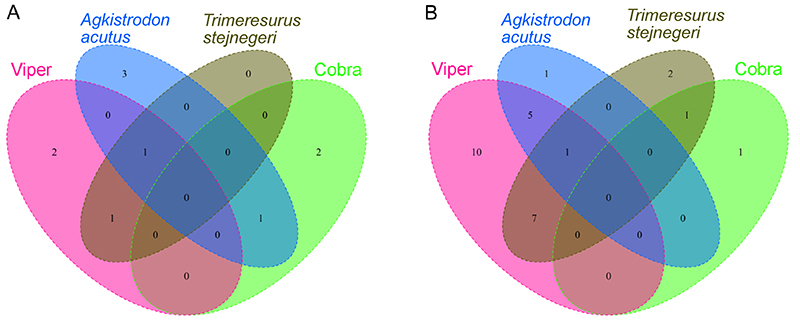
Venn diagrams depicting the distribution of upregulation and downregulation of the 38 differential abundance proteins (DAPs) with statistical significance. **(A)** The overlap of significantly upregulated protein in the four snakebite subgroups as compared to the healthy control group. **(B)** The overlap of significantly downregulated proteins in the four snakebite subgroups as compared to the healthy control group.

To investigate the differential expression of proteins in the snakebite groups and the healthy control group, four subgroups on the basis of snake species were individually analyzed to identify differential abundance proteins (DAPs) as compared with those in the healthy control group. The distributions of upregulation and downregulation of 93 DAPs in the four snakebite subgroups are shown in Additional file 3. Collectively, 38 proteins were expressed in groups representing two snake species and selected as significant candidates with a *p*-value < 0.05 and |log2fold change| > 1. Of these DAPs, 14 proteins were upregulated and 28 were downregulated relative to the peptide intensities of the healthy control group in Additional file 3. A Venn diagram depicted the overlap of 38 DAPs from the identified serum proteins between the snakebite groups and the healthy control group (Figure 2). Correspondingly, subgroup comparison found that 55 proteins, constituting 59% of total DAPs, displayed no expression in one of the snakebite subgroups and the healthy control group in Additional file 3. 

**Figure 3.**
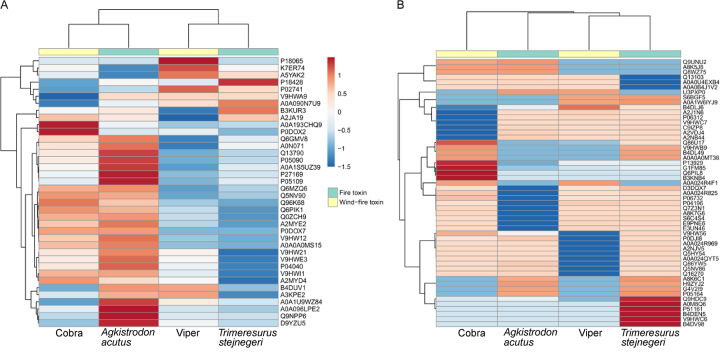
A biological heat map of DAP clustering based on fold changes between each snakebite subgroup and the healthy control group. **(A)** Thirty-eight proteins with statistically significant differences in expression were shown. **(B)** Fifty-five proteins displayed no expression in one group by subgroup comparisons. The color depth from blue to red represents the level of change of protein abundance; whereas blue and red represent, respectively, proteins with decreased and increased expression relative to the healthy control group.

Further comparisons between the four snakebite subgroups were detailed in a heat map (Figure 3). Unsupervised hierarchical clustering revealed different protein expression profiles among the four subgroups of snake species. However, no strong clustering was observed to separate wind-fire toxin and fire toxin (Figures 3A and 3B). Given the low overlap and lack of strong clustering of proteins between the four snakebite subgroups, great care should be taken in defining biomarker discovery for snakebites.

### Functional categorization of proteins identified in the serum

**Figure 4.**
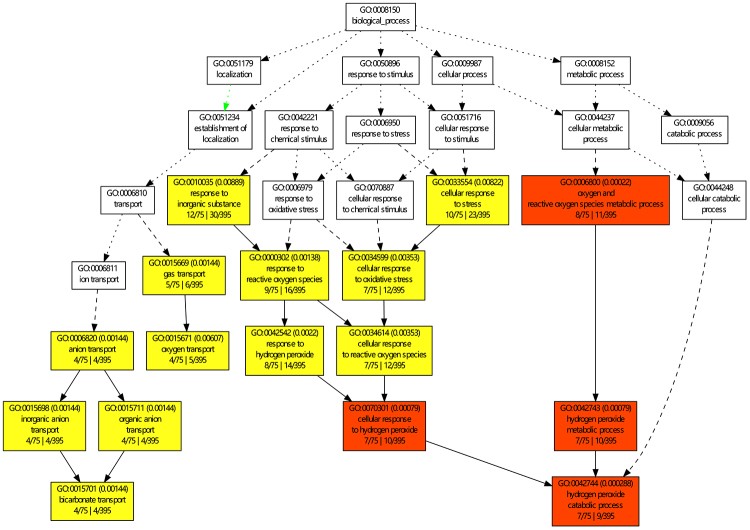
Gene Ontology (GO) enrichment analysis for venom-induced DAPs. GO terms were categorized by their associated biological processes as shown. All statistically significant categories are represented by color nodes, with those that are reddish representing a higher value of significance. The nodes of significant terms were defined as those with an adjusted p-value of less than 0.05. Functional enrichment of bicarbonate/oxygen transport and hydrogen peroxide catabolic process were tightly correlated to reactive oxygen species (ROS).

To identify potential molecular processes associated with serum proteins that are characteristic of snake venom-induced toxicity, all 474 proteins were categorized by their Gene Ontology (GO) terms. The GO annotations identified 395 proteins that were assigned to GO terms. GO analysis revealed that the biologic processes included defense/immunity response, cell motility, wounding healing, vesicle-mediated transport, lipid and gas transport, coagulation, protein metabolic process, and more (Additional file 4). The DAPs were categorized into fewer biological processes, including bicarbonate/oxygen transport and hydrogen peroxide catabolic process (Figure 4). The molecular function classes included carbon-oxygen lyase activity and heme binding. 

### The interaction network among proteins and chemicals

**Figure 5.**
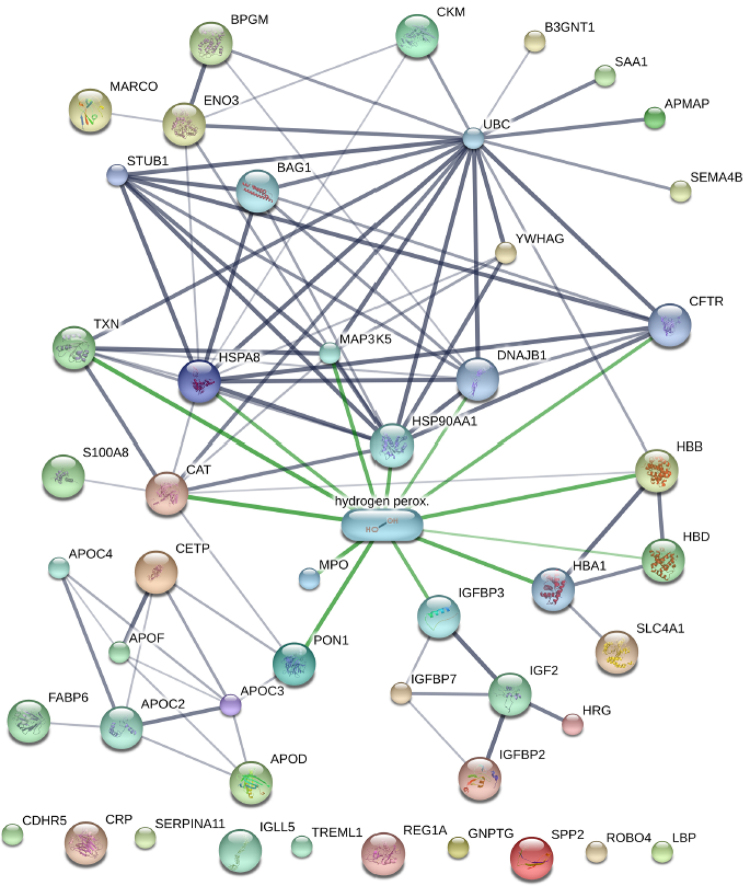
The network of protein and drug compound interactions. Thirty DAPs were found to be directly or indirectly associated with hydrogen peroxide. Colored nodes represent query DAPs, and small nodes represent those with an unknown 3D structure. Large nodes represent proteins with a known or predicted 3D structure. The weight of edges indicates the confidence score, wherein a thicker line indicates stronger association. Protein-protein interactions are represented by a gray edge, while chemical-protein interactions are depicted in green.

To understand the complex pathogenesis induced by snake venoms, the network of interacting proteins and drug compounds was constructed from the STITCH database [26]. Both chemicals and proteins were taken as nodes, and the linkage was taken as edges assigned weight scores to measure the strength of the association. In the constructed network of DAPs, we found one candidate compound, hydrogen peroxide, was highly associated with many DAPs (Figure 5). Forty-one proteins out of the 93 DAPs were matched with annotation in STITCH. In Additional file 5, 30 proteins of the DAPs showed complex interactions with 9 other proteins from STITCH, including BAG1, CFTR, DNAJB1, HBA1, HSP90AA1, IGFBP2, MAP3K5, STUB1, and UBC. One protein cluster consists of several apolipo proteins, such as APOC2, APOC3, APOC4, APOD, and APOF, which are associated with lipid metabolism and transport. Another cluster, including IGF2, IGFBP2, IGFBP3, IGFBP7, and HRG, is highly related to IGF-mediated growth. The third cluster, including HBA1, HBB, HBD, and SLC4A1, was associated with oxygen transport. The largest cluster was the ubiquitin mediated network, associated with innate immunity, protein degradation, and autophagy to eliminate toxic protein aggregates.

## Discussion

Snakebites from highly venomous species can be fatal [4, 28]. It is often difficult to find the appropriate antivenom for optimal clinical management, due to the misidentification of the snake species. Previously, there was a lack of careful studies investigating the protein profiles of the serum from snakebite patients. Thus, we used label-free protein expression profiling to identify potential protein biomarkers in the serum of patients bitten by four different snake species. We found that one candidate compound, hydrogen peroxide, was highly associated with many proteins in the interaction network of proteins and drug compounds.

The label-free method is an effective technique for the simultaneous identification and quantification of thousands of proteins, providing potential biomarkers in a highly focused pool [29, 30]. In this study, we quantified 735 non-redundant proteins in serum by LC-MS/MS analysis. Due to interpersonal variability, the expression proteins were qualified to satisfy the condition of at least two non-null intensities in replicates of each subgroup. This resulted in 474 unique proteins that showed high reproducibility between snakebite patients and the healthy control group. The distinct reduction in the number of shared proteins may result from biased estimates in the clinic in identifying similar snakes [31]. However, the wind-fire toxin patients that were bitten by vipers and cobras are distinguished from the fire toxin patients bitten by *Agkistrodon acutus* and *Trimeresurus stejnegeri* in TCM. Based on serum protein expression profiles, we observed a significant distinction in PCA and hierarchical clustering between the wind-fire toxin and fire toxin groups (Figure 1B and 1C). We observed two obvious clusters of significantly upregulated proteins, presented in Figure 1C. Therefore, exploring differences in the serum protein expression profile is effective to distinguish snakebites, as well as for biomarker and antivenom discovery. 

Discovering the pathways the identified serum proteins are associated with offers an opportunity for biomarker discovery. We found that the venom-induced toxicity impacted defense/immunity response, cell motility, wounding healing, and especially oxidative stress response. DAPs were associated with the hydrogen peroxide catabolic process, carbon-oxygen lyase activity, and heme binding. Previous studies have demonstrated that venom-induced toxicity elevates reactive oxygen species (ROS) and hydrogen peroxide levels, implying potential cytotoxicity of venom [11, 32]. The compound hydrogen peroxide is an important component of ROS, which regulates many cellular functions in limited concentrations, but can also cause damage of cellular organelles, DNA, and proteins when present in excessive concentrations, an effect known as oxidative stress [33]. The interaction network of proteins and drug compounds showed that hydrogen peroxide was highly associated with many DAPs (Figure 5). These interacting DAPs were grouped into four clusters, including lipid metabolism and transport, IGF-mediated growth, oxygen transport, and innate immunity. The venom-induced toxicity activates various oxidases and respiratory burst due to inflammation [34]. Phospholipases A2 (PLA2s) are abundant components of venom in many snake species, and have the effect of blocking neuromuscular transmission and inducing acute muscle damage [35]. However, the physiological roles of PLA2s remain unknown. We discovered that a large cluster in the protein interaction network is concentrated in the innate ubiquitin-proteasome. Protein degradation may potentially be the cellular targets of PLA2s. The molecular composition of venom and serum from snakebite patients may be the most direct and credible way to evaluate the molecular mechanisms underlying the venom-induced toxicity process.

Comparison of DAPs in snakebites from different snake species identifies potential biomarkers and provides theoretical support for TCM on the molecular level. A few proteins participating in the networks were upregulated in the serum of the fire toxin group as compared to the wind-fire toxin group, such as TXN (Thioredoxin), CETP (Cholesteryl ester transfer protein), IGFBP7 (Insulin-like growth factor binding protein 7), MPO (Myeloperoxidase), and SLC4A1 (Solute carrier family 4). One protein, YWHAG (Tyrosine 3-monooxygenase/tryptophan 5-monooxygenase activation protein), was only upregulated in the B subgroup (vipers), while one protein IGF-2 (Insulin-like growth factor 2) was only upregulated in the C subgroup (cobras). Two proteins, FABP6 (Fatty acid binding protein 6) and B3GNT1 (Beta-1,3-N-acetylglucosaminyltransferase 1), were only upregulated in the D subgroup (*Agkistrodon acutus*), while four proteins, HSPA8 (Heat shock 70kDa protein 8), CKM (Creatine kinase), SEMA4 (semaphorin 4B), and SAA1 (serum amyloid A1) were only unexpressed in the E subgroup (*Trimeresurus stejnegeri*). These potential protein biomarkers well illustrated in toxin classification of TCM theory, and provided more biomarkers for identification of snakebite by different snake species. Importantly, the combined DAPs from four snake species suggested that they were mainly involved in the interaction network of proteins and hydrogen peroxide. Envenomation has been shown to induce redox status imbalance in a few snakes [36]. Some of these upregulated proteins, such as TXN, CETP, IGFBP7, and IGF-2, have been found to coincide with an increase of reactive oxygen species, which could act as antioxidants by reducing glutathione ratio (GSSG/GSH) [37-40]. Thus, the DAPs associated with oxidative stress and antioxidant action could be regarded as prior consideration for experimental validation in the future.

## Conclusion

Five proteins (TXN, CETP, IGFBP7, MPO, and SLC4A1) were potential biomarkers to distinguish fire toxin and wind-fire toxin in classification of TCM theory, and a few proteins could identify snakebite of different snake species. These potential protein biomarkers were connected into the interaction network of proteins and hydrogen peroxide, which indicated that the venom-induced toxicity of snakebites was associated with oxidative stress and antioxidant defense. In the future, these potential biomarkers will need to be screened in a larger cohort of patients for clinical application.

## Supplementary material

The following online material is available for this article:

Additional file 1. Peptide identification reported using MaxQuant analysis.

Additional file 2. Protein quantification with LFQ intensity profiles obtained after normalization by the MaxLFQ algorithm.

Additional file 3. The distribution of 93 differential abundance proteins (DAPs) among each snakebite subgroup and the healthy control group. Upregulation and downregulation of 38 DAPs are statistically significant in expression. Expression and no expression of 55 proteins represent only detection in one subgroup. The total number is larger than the number of DAPs because of the overlap of DAPs in the four snakebite subgroups as compared to the healthy control group. The columns “B *vs*. A”, “C *vs*. A”, “D *vs*. A”, and “E *vs*. A” indicated the snakebite subgroup comparison with the healthy control group.

Additional file 4. Gene Ontology (GO) analysis of all 474 identified proteins revealed the following significantly enriched terms in biological processes: defense/immunity response, cell motility, wounding healing, vesicle-mediated transport, lipid and gas transport, coagulation, protein metabolic process, and others.

Additional file 5. List of the differential abundance proteins (DAPs) participating in the network of protein and drug compound interactions.

## References

[B1] Gutierrez JM, Calvete JJ, Habib AG, Harrison RA, Williams DJ, Warrell DA (2017). Snakebite envenoming. Nat Rev Dis Primers.

[B2] Bhaumik S (2013). Snakebite: a forgotten problem. BMJ.

[B3] Vongphoumy I, Phongmany P, Sydala S, Prasith N, Reintjes R, Blessmann J (2015). Snakebites in two rural districts in Lao PDR: Community-based surveys disclose high incidence of an invisible public health problem. PLoS Negl Trop Dis.

[B4] Alirol E, Sharma SK, Bawaskar HS, Kuch U, Chappuis F (2010). Snake bite in South Asia: a review. PLoS Negl Trop Dis.

[B5] Chippaux JP, Massougbodji A, Habib AG (2019). The WHO strategy for prevention and control of snakebite envenoming: a sub-Saharan Africa plan. J Venom Anim Toxins incl Trop Dis.

[B6] Chippaux JP (2017). Snakebite envenomation turns again into a neglected tropical disease!. J Venom Anim Toxins incl Trop Dis.

[B7] Fan QS, Wang SY, Qiu W (2005). Studies on treatment of Viper bites in Chineese military.).

[B8] Gren ECK, Kitano ES, Andrade-Silva D, Iwai LK, Reis MS, Menezes MC (2019). Comparative analysis of the high molecular mass subproteomes of eight Bothrops snake venoms. Comp Biochem Physiol Part D.

[B9] Fatima L, Fatah C (2014). Pathophysiological and Pharmacological Effects of Snake Venom Components: Molecular Targets. J Clin Toxicol.

[B10] Mehta SR, Sashindran VK (2002). Clinical Features And Management Of Snake Bite. Med J Armed Forces India.

[B11] Tan CH, Wong KY, Chong HP, Tan NH, Tan KY (2019). Proteomic insights into short neurotoxin-driven, highly neurotoxic venom of Philippine cobra (Naja philippinensis) and toxicity correlation of cobra envenomation in Asia. J Proteom.

[B12] Fox JW, Serrano SM (2009). Timeline of key events in snake venom metalloproteinase research. J Proteomics.

[B13] Patra A, Chanda A, Mukherjee AK (2019). Quantitative proteomic analysis of venom from Southern India common krait (Bungarus caeruleus) and identification of poorly immunogenic toxins by immune-profiling against commercial antivenom. Expert Rev Proteomics.

[B14] Kittigul L, Ratanabanangkoon K (1993). Reverse passive hemagglutination tests for rapid diagnosis of snake envenomation. J Immunoassay.

[B15] Maduwage K, O'Leary MA, Isbister GK (2014). Diagnosis of snake envenomation using a simple phospholipase A2 assay. Sci Rep.

[B16] Deng T, Ergil K (1999). Practical diagnosis in traditional Chinese medicine.

[B17] Sun K, Fan J, Han J (2015). Ameliorating effects of traditional Chinese medicine preparation, Chinese materia medica and active compounds on ischemia/reperfusion-induced cerebral microcirculatory disturbances and neuron damage. Acta Pharm Sin B.

[B18] Tu C, Rudnick PA, Martinez MY, Cheek KL, Stein SE, Slebos RJC (2010). Depletion of abundant plasma proteins and limitations of plasma proteomics. J Proteome Res.

[B19] Agilent Technologies (2007). Agilent Human 14 Multiple Affinity Removal System Columns for the Fractionation of High-Abundant Proteins from Human Proteomic Samples.

[B20] Laemmli UK (1970). Cleavage of structural proteins during the assembly of the head of bacteriophage T4. Nature.

[B21] Candiano G, Bruschi M, Musante L, Santucci L, Ghiggeri GM, Carnemolla B (2004). Blue silver: a very sensitive colloidal Coomassie G-250 staining for proteome analysis. Electrophoresis.

[B22] Bhosale SD, Moulder R, Kouvonen P, Lahesmaa R, Goodlett DR (2017). Mass spectrometry-based serum proteomics for biomarker discovery and validation. Methods Mol Biol.

[B23] Cox J, Neuhauser N, Michalski A, Scheltema RA, Olsen JV, Mann M (2014). Andromeda: a peptide search engine integrated into the MaxQuant environment. J Proteome Res.

[B24] Cox J, Mann M (2008). MaxQuant enables high peptide identification rates, individualized p.p.b.-range mass accuracies and proteome-wide protein quantification. Nat Biotechnol.

[B25] Metsalu T, Vilo J (2015). ClustVis: a web tool for visualizing clustering of multivariate data using Principal Component Analysis and heatmap. Nucleic Acids Res.

[B26] Szklarczyk D, Santos A, von Mering C, Jensen LJ, Bork P, Kuhn M (2015). STITCH 5: augmenting protein-chemical interaction networks with tissue and affinity data. Nucleic Acids Res.

[B27] Szklarczyk D, Franceschini A, Wyder S, Forslund K, Heller D, Huerta-Cepas J (2015). STRING v10: protein-protein interaction networks, integrated over the tree of life. Nucleic Acids Res.

[B28] Gutierrez JM (2020). Snakebite envenoming from an Ecohealth perspective. Toxicon X.

[B29] Higgs RE, Knierman MD, Gelfanova V, Butler JP, Hale JE (2008). Label-free LC-MS method for the identification of biomarkers. Methods Mol Biol.

[B30] Qian WJ, Jacobs JM, Liu T, Camp DG, Smith RD (2006). Advances and challenges in liquid chromatography-mass spectrometry-based proteomics profiling for clinical applications. Mol Cell Proteomics.

[B31] Wuster W, Allum CS, Bjargardottir IB, Bailey KL, Dawson KJ, Guenioui J (2004). Do aposematism and Batesian mimicry require bright colours? A test, using European viper markings. Proc Biol Sci.

[B32] Santhosh MS, Sundaram MS, Sunitha K, Kemparaju K, Girish KS (2013). Viper venom-induced oxidative stress and activation of inflammatory cytokines: a therapeutic approach for overlooked issues of snakebite management. Inflamm Res.

[B33] Marnett LJ (2000). Oxyradicals and DNA damage. Carcinogenesis.

[B34] Sampaio SC, Sousa-e-Silva MC, Borelli P, Curi R, Cury Y (2001). Crotalus durissus terrificus snake venom regulates macrophage metabolism and function. J Leukoc Biol.

[B35] Gutierrez JM, Lomonte B (2013). Phospholipases A2: unveiling the secrets of a functionally versatile group of snake venom toxins. Toxicon.

[B36] Sachetto ATA, Rosa JG, Santoro ML (2018). Rutin (quercetin-3-rutinoside) modulates the hemostatic disturbances and redox imbalance induced by Bothrops jararaca snake venom in mice. PLoS Negl Trop Dis.

[B37] Zengin S, Al B, Yarbil P, Taysi S, Bilinc H, Yildirim C (2013). Oxidant/antioxidant status in cases of snake bite. J Emerg Med.

[B38] Boekholdt SM, Kuivenhoven JA, Hovingh GK, Jukema JW, Kastelein JJ, van Tol A (2004). CETP gene variation: relation to lipid parameters and cardiovascular risk. Curr Opin Lipidol.

[B39] Cagnone GL, Sirard MA (2013). Transcriptomic signature to oxidative stress exposure at the time of embryonic genome activation in bovine blastocysts. Mol Reprod Dev.

[B40] Aquilano K, Baldelli S, Ciriolo MR (2014). Glutathione: new roles in redox signaling for an old antioxidant. Front Pharmacol.

