# Supplementary material for: Oxidative stress and antioxidant defense in detoxification systems of snake venom-induced toxicity
Source: J Venom Anim Toxins Incl Trop Dis. 2020 Oct 19;26:e20200053. doi: 10.1590/1678-9199-JVATITD-2020-0053 (PMC7574533; doi:10.1590/1678-9199-JVATITD-2020-0053)
Supplement: Additional file 3. [file 1678-9199-jvatitd-26-e20200053-s3.pdf]

J Venom Anim Toxins incl Trop Dis

https://doi.org/10.1590/1678-9199-JVATITD-2020-0053

Supplementary Material to "Oxidative stress and antioxidant defense in detoxification systems of snake venom-induced toxicity"

Additional file 3. The distribution of 93 differential abundance proteins (DAPs) among each snakebite subgroup and the healthy control group. Upregulation and downregulation of 38 DAPs are statistically significant in expression. Expression and no expression of 55 proteins represent only detection in one subgroup. The total number is larger than the number of DAPs because of the overlap of DAPs in the four snakebite subgroups as compared to the healthy control group. The columns "B *vs.* A", "C *vs.* A", "D *vs.* A", and "E *vs.* A" indicated the snakebite subgroup comparison with the healthy control group.

	**B *vs.* A**	**C *vs.* A**	**D *vs.* A**	**E *vs.* A**
Upregulation	4	3	5	2
Downregulation	23	2	7	11
Expression	26	20	33	24
No expression	8	11	9	10
**Total**	**61**	**36**	**54**	**37**

A: healthy control; B: vipers; C: cobras; D: *Agkistrodon acutus*; E: *Trimeresurus stejnegeri.*
