# Supplementary material for: Oxidative stress and antioxidant defense in detoxification systems of snake venom-induced toxicity
Source: J Venom Anim Toxins Incl Trop Dis. 2020 Oct 19;26:e20200053. doi: 10.1590/1678-9199-JVATITD-2020-0053 (PMC7574533; doi:10.1590/1678-9199-JVATITD-2020-0053)
Supplement: Additional file 4. [file 1678-9199-jvatitd-26-e20200053-s4.pdf]

J Venom Anim Toxins incl Trop Dis

https://doi.org/10.1590/1678-9199-JVATITD-2020-0053

Supplementary Material to “Oxidative stress and antioxidant defense in detoxification systems of snake venom-induced toxicity”

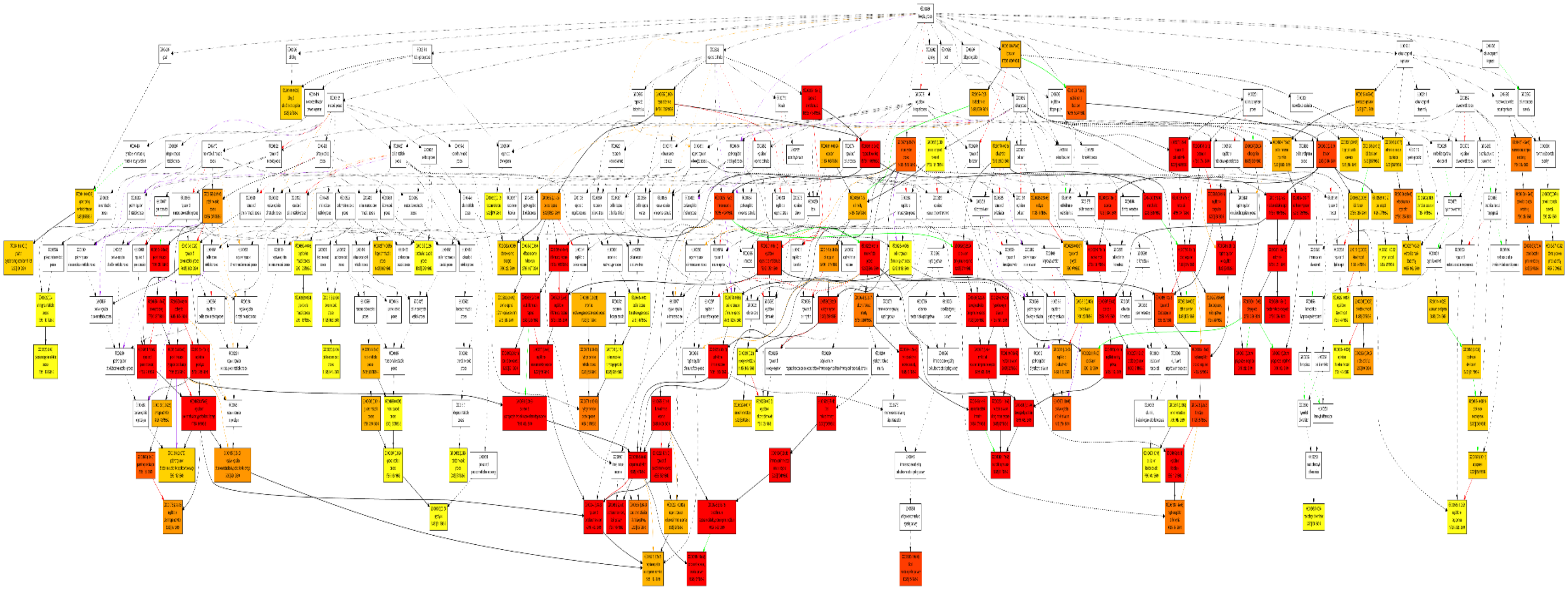

Additional file 4. Gene Ontology (GO) analysis of all 474 identified proteins revealed the following significantly enriched terms in biological processes: defense/immunity response, cell motility, wounding healing, vesicle-mediated transport, lipid and gas transport, coagulation, protein metabolic process, and others.
