# Supplementary material for: Oxidative stress and antioxidant defense in detoxification systems of snake venom-induced toxicity
Source: J Venom Anim Toxins Incl Trop Dis. 2020 Oct 19;26:e20200053. doi: 10.1590/1678-9199-JVATITD-2020-0053 (PMC7574533; doi:10.1590/1678-9199-JVATITD-2020-0053)
Supplement: Additional file 5. [file 1678-9199-jvatitd-26-e20200053-s5.pdf]

J Venom Anim Toxins incl Trop Dis

https://doi.org/10.1590/1678-9199-JVATITD-2020-0053

Supplementary Material to "Oxidative stress and antioxidant defense in detoxification systems of snake venom-induced toxicity"

Additional file 5. List of the differential abundance proteins (DAPs) participating in the network of protein and drug compound interactions.

Node links	Protein IDs	Gene names	Wind-fire toxin		Fire toxin		Protein names
			B/A	C/A	D/A	E/A	
12	E9PNE6	HSPA8	23.94	24.34	24.05	0.00	Heat shock 70kDa protein 8
9	P04040	CAT	-1.20	-0.93	-0.36	-1.94	Catalase
7	H9ZYJ2	TXN	0.00	0.00	25.61	25.82	Thioredoxin
6	P13929	ENO3	26.98	26.21	26.17	25.83	Enolase 3
6	A3KPE2	APOC3	-1.43	-1.88	-1.46	-2.14	Apolipoprotein C-III
5	K7ER74	APOC2	-1.27	-1.62	-1.96	-1.63	Apolipoprotein C-II
5	D9YZU5	HBB	-1.56	-1.62	-1.44	-1.60	Hemoglobin, beta
4	Q13790	APOF	0.46	1.35	2.11	0.69	Apolipoprotein F
4	P27169	PON1	0.54	1.10	2.36	0.79	Paraoxonase 1
4	E3UN46	IGF2	0.42	1.15	0.46	-28.01	Insulin-like growth factor 2
4	B3KNB4	YWHAG	23.33	0.00	0.00	0.00	Tyrosine 3-monooxygenase/tryptophan 5-monooxygenase activation protein, gamma polypeptide
4	A8K6C1	CETP	0.00	0.00	28.14	26.13	Cholesteryl ester transfer protein, plasma
3	Q16270	IGFBP7	25.07	0.00	25.11	25.75	Insulin-like growth factor binding protein 7
3	P06732	CKM	29.28	25.48	27.11	0.00	Creatine kinase
3	P05090	APOD	0.54	1.02	1.47	0.68	Apolipoprotein D
3	B3KUR3	BPGM	-1.08	-0.32	-0.18	0.27	2,3-bisphosphoglycerate mutase
3	A5YAK2	APOC4	-1.03	-1.65	-2.10	-1.32	Apolipoprotein C-IV
3	A0N071	HBD	-1.81	-1.46	-1.31	-1.56	Hemoglobin, delta
2	P18065	IGFBP2	1.98	1.38	1.33	1.31	Insulin-like growth factor binding protein 2
1	Q9HDC9	APMAP	25.84	25.69	26.47	25.93	Adipocyte plasma membrane associated protein
1	Q7Z3N1	SEMA4B	24.71	24.48	24.52	0.00	Semaphorin 4B
1	P51161	FABP6	0.00	0.00	24.94	0.00	Fatty acid binding protein 6
1	P0DJI8	SAA1	27.53	0.00	29.77	31.52	Serum amyloid A1
1	P05164	MPO	0.00	0.00	25.94	24.87	Myeloperoxidase
1	P05109	S100A8	1.11	1.46	2.40	1.42	S100 calcium binding protein A8
1	P04196	HRG	0.23	3.07	2.24	-28.42	Histidine-rich glycoprotein
1	G4V2I9	SLC4A1	0.00	0.00	28.08	27.43	Solute carrier family 4
1	D3DQX7	SAA1	25.05	24.25	27.93	0.00	Serum amyloid A1
1	B4DLJ6	MARCO	25.57	26.32	26.10	25.91	Macrophage receptor with collagenous structure
1	B4DEN5	B3GNT1	0.00	0.00	23.77	0.00	Beta-1,3-N-acetylglucosaminyltransferase 1

Protein IDs: Uniprot IDs of the protein group; gene names: official gene name; protein names: the name of the identified protein; node links: the edge numbers linked to the protein node. A: healthy control; B: vipers; C: cobras; D: *Agkistrodon acutus*; E: *Trimeresurus stejnegeri*. B/A, C/A, D/A, and E/A: log_2FC.
